# Disaster Risk from Extreme Natural Events Influences Scientific Production on Riparian Forest Carbon More than Socioeconomic Variables

**DOI:** 10.1007/s00267-026-02408-1

**Published:** 2026-02-24

**Authors:** Julia Isabella de Matos Rodrigues, Lucas Sérgio de Sousa Lopes, Victor Pereira de Oliveira, Joathan Cipriano Castro, Hiago Felipe Cardoso Pacheco, Gracialda Costa Ferreira, Francisco de Assis Oliveira, Walmer Bruno Rocha Martins

**Affiliations:** 1 Federal Rural University of Amazonia, Belém, Pará Brazil; 2 Federal Rural University of Amazonia, Capitão Poço, Pará Brazil; 3 National Institute for Amazonian Research, Constelação Cruzeiro do Sul Avenue, Manaus, Amazonas Brazil; 4 Vale Institute of Technology Sustainable Development, Belém, Pará Brazil; 5 Federal University of Viçosa, Viçosa, Minas Gerais Brazil

**Keywords:** climate change socioeconomic variables collaborative networks

## Abstract

Riparian forests are key ecosystems for mitigating the adverse effects of climate change due to their high potential for carbon sequestration and storage. However, the dynamics of scientific production on carbon in these ecosystems remain poorly understood. This study conducted a bibliometric analysis of publications indexed in the Scopus and Web of Science databases, aiming to identify temporal trends, geographic distribution, collaboration networks, and socioeconomic factors associated with scientific production on the topic. Descriptive statistics, co-authorship network analysis, and a negative binomial regression model were used to assess the influence of variables such as GDP, life expectancy, literacy rate, greenhouse gas (GHG) emissions growth, and disaster risk from natural extreme events on global scientific production. A total of 921 studies were cataloged, with the United States (*n* = 92), Indonesia (*n* = 85), and China (*n* = 82) showing the highest number of studies. The United States led international collaboration. A significant positive effect of disaster risk was observed on the number of publications (β = 0.0524; *p* < 0.001), indicating that greater exposure to extreme natural events is associated with increased research on riparian forest carbon. GDP also showed a statistically significant association with scientific production, although with a weak effect, reflecting its role as a structural capacity factor rather than a primary driver. Other socioeconomic variables were not statistically significant. Therefore, scientific production appears to be governed by a dual mechanism, combining structural research capacity, represented by GDP, and contextual environmental pressure, represented by disaster risk.

## Introduction

The global climate crisis and the intensification of ecosystem degradation have heightened the urgency for effective strategies in conservation, mitigation, and environmental restoration. Among the ecosystems most relevant to sustainability, riparian forests stand out as key areas, because these ecosystems consist of strips of natural vegetation adjacent to watercourses, functioning as transitional zones between terrestrial and aquatic environments (Verdonschot and Verdonschot 2023). Riparian forests provide multiple ecosystem services, including water regulation, biodiversity protection, and geomorphological stability (Dinca et al. 2025). In addition to these services, riparian forests are considered key ecosystems for climate change mitigation due to their high productivity and, consequently, their substantial capacity for carbon storage. These forests can accumulate between 88 and 202 Mg C ha^−1^ in the aboveground biomass of tropical riparian systems (Elarayán et al. 2015; Pasion et al. 2021), and up to 489.16 Mg C ha^−1^ in organic soil carbon of riparian temperate forests (Ofosu et al. 2022). Furthermore, riparian soils are often characterized by high organic matter content, resulting from constant litterfall deposition and the oxidation of moist soils (Liu et al. 2024).

Despite their ecological importance, riparian forests have undergone accelerated degradation, fragmentation, and conversion in recent decades due to the expansion of anthropogenic activities such as intensive agriculture, mining, and unplanned urbanization, leading to significant carbon stock losses (Lima et al. 2022; Rojas-Castillo et al. 2024). Older riparian forests store four times more carbon than younger ones (Giese et al. 2003), and their conversion into monocultures has led to a 14% reduction in soil carbon storage, as observed in Indonesia (Hennings et al. 2021). In light of this scenario, public policies and forest restoration programs have increasingly recognized riparian forests as essential green infrastructure for climate resilience and water security (Shah and Race 2024). However, the capacity of countries to generate scientific knowledge that supports such policies and restoration efforts is not uniform and is strongly conditioned by broader socioeconomic and environmental contexts (Jack et al. 2021). National differences in economic resources, human development, educational level, environmental pressure, and exposure to climate-related hazards can influence research infrastructure, funding availability, and scientific priorities. In this sense, variables such as Gross Domestic Product (GDP), life expectancy, literacy rates, greenhouse gas (GHG) emission growth, and the risk of natural disasters caused by extreme events may shape both the volume and focus of scientific output on environmental issues, including riparian forest research (Adams et al. 2016; Lemarié and Marette 2022).

Previous studies synthesizing information on carbon stocks have largely focused on intra-ecosystem factors influencing carbon storage, such as physical barriers (Sutfin et al. 2016). However, it is still important to understand how scientific knowledge on riparian forest carbon has developed across countries and regions, as there is limited understanding of how socioeconomic drivers and environmental risks may influence the global production of knowledge on riparian forests and their role in climate change mitigation. In this context, examining global patterns of scientific production on riparian forest carbon can support evidence-based public policies and inform more effective investments in conservation. Therefore, this study examines how external factors, particularly socioeconomic (GDP, literacy rate, and life expectance) and environmental variables (GHC emissions and disaster risk from natural events), are associated with scientific production related to riparian forests, contributing to a clearer understanding of differences in knowledge production and environmental response capacity among countries.

Given this context, bibliometric analysis emerges as a strategic tool for mapping scientific production patterns, identifying knowledge gaps, and tracking the evolution of international collaborations, methodological approaches, and emerging research themes in the field (Maretti et al. 2019; Martins et al. 2020; Ellili 2023). Accordingly, this study aimed to synthesize the scientific literature on riparian forests and investigate whether scientific production is associated with the socioeconomic and environmental characteristics of countries. Specifically, the study sought to: a) identify temporal trends in publications, leading countries, authors, and research topics related to riparian forests; b) map international collaboration networks; and c) analyze potential associations between socioeconomic and environmental variables and scientific production on riparian forests.

## **Methods**

### Search Strategy and Study Selection

**Fig. 1:**
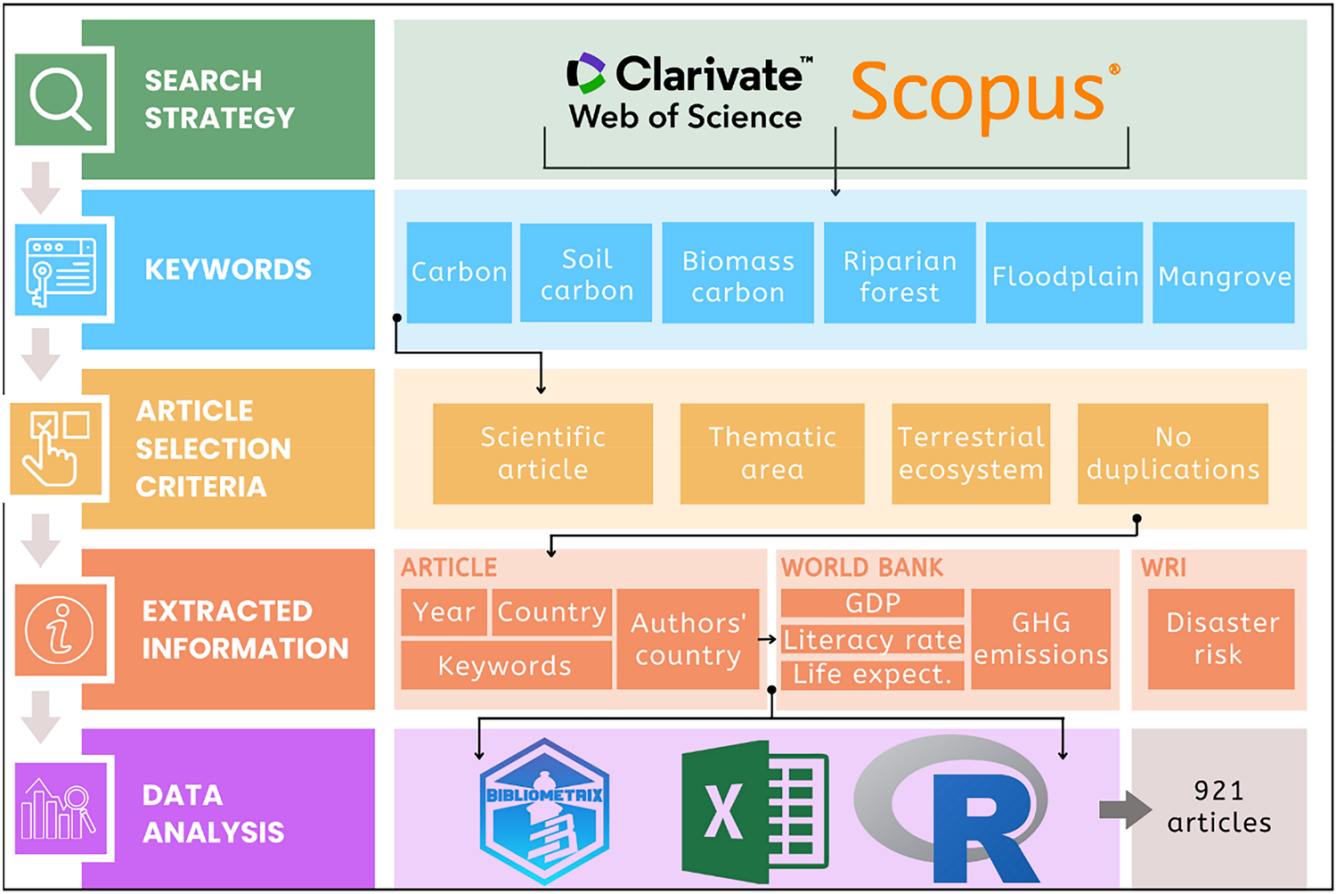
Procedures for selecting and collecting information on scientific production related to carbon stocks in riparian forests, as well as the socioeconomic indicators of the countries where the articles were published

Searches were conducted in the Scopus and Web of Science databases using a systematic keyword search: (“carbon” OR “soil carbon” OR “biomass carbon” AND “riparian forest” OR “floodplain” OR “mangrove”), targeting terms found in the title, abstract, or keywords. To be included in the analysis, studies were required to meet four criteria: a) be a peer-reviewed scientific article; b) be classified under subject areas related to forest sciences, agricultural sciences, environmental sciences, or related fields; c) be conducted in terrestrial ecosystems, excluding studies that focused exclusively on carbon in water or fauna; and d) not be a duplicate entry (Fig. 1).

The ecosystem-related terms “riparian forest,” “floodplain,” and “mangrove” were used to capture the main forest types occurring along fluvial and tidal gradients, ensuring broad coverage of riparian-related environments investigated in the literature. Carbon-related keywords (“carbon,” “soil carbon,” and “biomass carbon”) were included because carbon stocks and dynamics represent one of the most recurrent and integrative research themes in riparian forest studies, strongly linked to ecosystem functioning, climate regulation, and land-use change. The inclusion of subject areas such as forest sciences, agricultural sciences, and environmental sciences reflects the ecological and scientific focus of riparian forest research, through studies about vegetation structure, ecosystem dynamics, nutrient cycling, and environmental processes, highlighting the interdisciplinary integration of ecological and biogeochemical perspectives in the study of riparian forests.

To capture the entire historical series of scientific production on carbon stocks in riparian forests, no language filters or publication year restrictions were applied. Thus, all articles available up to February 2025, the date on which the searches were conducted, were considered. To limit the results to peer-reviewed scientific articles and ensure thematic relevance, document type (articles) and subject area filters provided by the search platforms were applied (forest sciences, agricultural sciences, environmental sciences, or related fields).

The screening process was carried out in two stages: I) reading of the title and abstract, and II) full-text reading. Full-text reading was performed only for studies that met the inclusion criteria during the first screening stage. Both stages were performed by a single reviewer to avoid subjectivity. To control duplication between databases, a unique identification code was created for each article, composed of the first six letters of the title, the first three letters of the country where the research was conducted, and the year of publication. Based on this code, a preliminary duplication check was performed. Articles were then organized in chronological order, allowing the identification of cases in which the same study had been published more than once in different years. In such cases, only the earliest version was retained, assuming it to be the original research.

### Data Extraction

From the selected scientific articles, the following information was extracted: year of publication, country where the study was conducted (when indicated), a listing of each article’s keywords, and authors’ institutional affiliations. In cases where studies were conducted in more than one country, each country was recorded separately, and the article was counted once for each country involved to adequately reflect multinational research efforts. We used authors’ institutional affiliations as they represent the institutional address reported at the time the research was conducted and the manuscript submitted, in accordance with journal guidelines. Accordingly, the country associated with each institutional affiliation was considered the formal location from which the research activity was carried out and it was employed as a proxy for identifying and characterizing international scientific collaboration.

Disaster risk from natural extreme events data were obtained from the 2025 World Risk Index –floods (https://weltrisikobericht.de/worldriskreport/). This variable was chosen because flooding is intrinsically linked to riparian forest dynamics, and extreme events can substantially impair the provision of ecosystem services in these systems. Moreover, floods are among the most frequent and impactful natural disasters worldwide (Frege et al. 2025). In addition, data on Gross Domestic Product (GDP), life expectancy, literacy rate, and greenhouse gas (GHG) emissions growth for each country, covering the time series from 1960 to 2023, were obtained using the WDI package (Arel**-**Bundock 2025) in the R software (R Development Core Team 2025), which compiles data from the World Bank (https://www.worldbank.org/ext/en/home).

The period from 1960 to 2023 corresponds to the full temporal coverage available for these indicators in the World Bank database and was not restricted to the publication period of the identified studies, allowing a comprehensive assessment of long-term socioeconomic and environmental trajectories. Life expectancy and literacy rate were included as complementary socioeconomic indicators representing overall human development, educational level, and institutional capacity, which may indirectly influence national research infrastructure, scientific investment, and knowledge production. GDP reflects a country’s aggregate economic capacity, which is more directly related to potential investment in research infrastructure, funding availability, and national scientific output, while GHG emissions growth represents the environmental and climate-related context potentially associated with research priorities and scientific production.

### Data Analysis

Scientific production over time was analyzed by annual publication counts, while country-level production was assessed using the total number of publications. Keywords were compiled and standardized, and their frequencies were calculated across all selected articles. The most frequent keywords were then visualized using a keyword cloud, highlighting both the 70 most frequent terms and the 15 most used keywords over the study period. International collaboration was evaluated based on authors’ institutional affiliations recorded in each article, enabling the creation of a country co-occurrence matrix using the igraph package (Csárdi et al. 2025) and graphical plotting with the ggraph package (Pedersen 2024). Furthermore, to investigate potential socioeconomic determinants of scientific production, a generalized linear model (GLM) was fitted, with the number of publications per country as the dependent variable and GDP, life expectancy, literacy rate, and greenhouse gas emissions growth as explanatory variables.

For model selection, a Poisson regression was initially fitted but showed high overdispersion (>15). To address this, a quasi-Poisson model, which automatically adjusts for dispersion, was tested; however, overdispersion persisted (>37). Consequently, a negative binomial regression model was fitted using the MASS package (Venables and Ripley 2002). The negative binomial model was validated through residual simulation with the DHARMa package (Hartig 2024). Model diagnostics included tests for dispersion and uniformity of simulated residuals based on the Kolmogorov–Smirnov test, as well as visual inspection of residuals plotted against fitted values to assess the presence of systematic structure (e.g., trends or heteroscedasticity). The marginal effects of explanatory variables on publication counts were interpreted using incidence rate ratios (exponentiated coefficients).

## Results

**Fig. 2:**
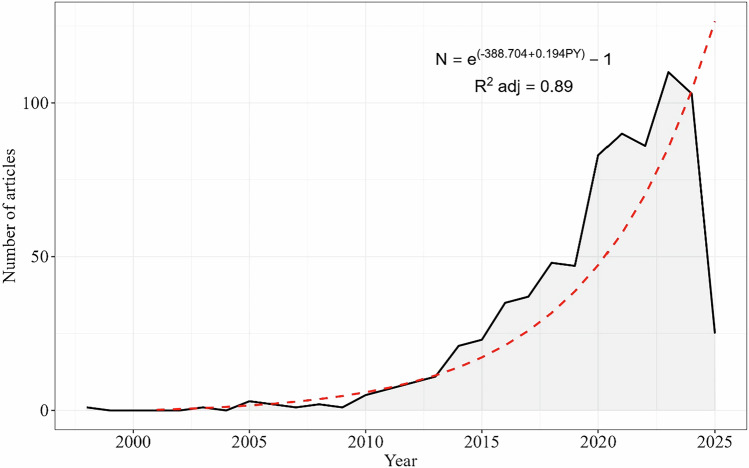
Exponential growth in the global number of published articles on carbon stocks in riparian forests. Data include all articles available up to February 2025

A total of 3162 scientific articles were found, but after exclusion criteria, 921 scientific articles on carbon stocks in riparian forests indexed in the Scopus and Web of Science database were compiled, with the earliest publication dating back to 1998. Between 1998 and 2009, the annual number of publications ranged from 0 to 3 articles, totaling only 11 publications over 12 years (Fig. 2). Starting in 2010, a growth trajectory began, with 196 articles published between 2010 and 2018, an 860% increase in the number of publications. In 2019, there was a slight decrease of 2.1%, with 47 articles published. However, in 2020, the number of publications nearly doubled compared to the previous year, reaching 83 articles. In the following years, the publication count remained high, with 90 articles recorded in 2021, 86 in 2022 (a 4.4% decrease), and the highest number in the historical series in 2023, with 110 publications. In 2024, there was a slight decrease of 6.4%, totaling 103 publications. For 2025, considering only January and February, 25 articles have already been identified (Fig. 2).

**Fig. 3:**
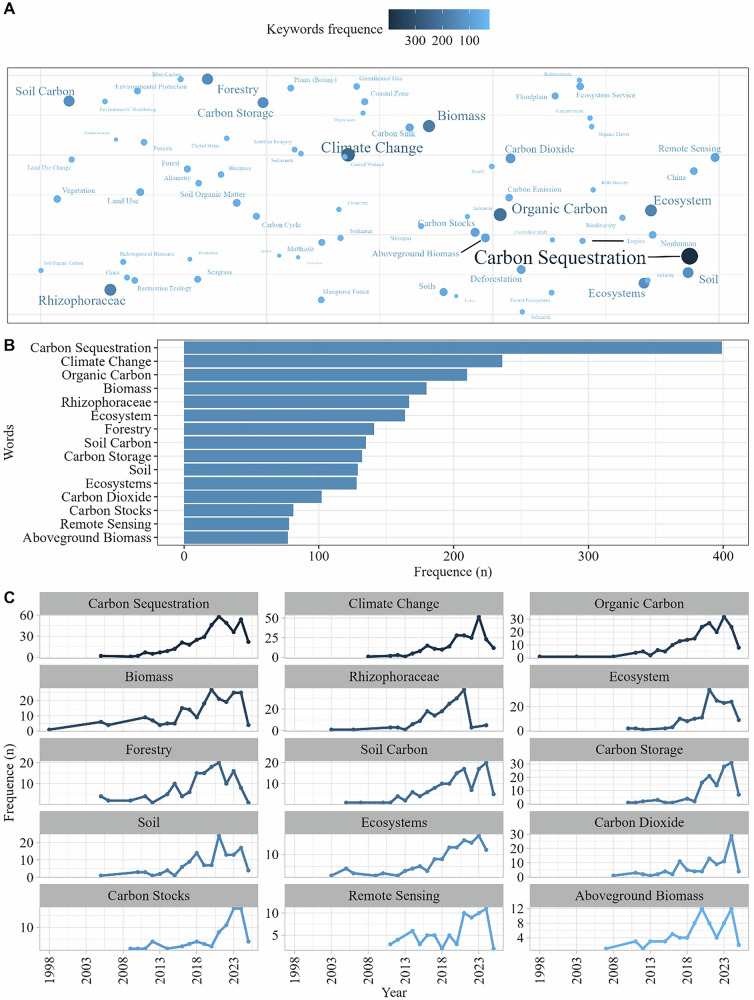
Keyword cloud showing the 70 most frequent keywords (**A**) and the 15 most used keywords (**B**) with temporal trend (**C**) in scientific articles published globally on carbon stocks in riparian forests over the years. Size and color of bubble signify research output frequence. Data include all articles available up to February 2025

The keywords “carbon sequestration” (*n* = 399), “climate change” (*n* = 236), and “organic carbon” (*n* = 210) were the most frequent across the articles. Other prominent terms included “biomass” (*n* = 180), “Rhizophoraceae” (*n* = 167), “forestry” (*n* = 141), “soil carbon” (*n* = 135), “carbon storage” (*n* = 132), “soil” (*n* = 129), and “carbon dioxide” (*n* = 102). Additional recurring terms were “carbon stocks” (*n* = 81) and “land use” (*n* = 56). The keyword “remote sensing” appeared as the 14th most frequent keyword (*n* = 78; Fig. 3B), and the first occurrence was in 2011 (Fig. 3D). Terms related to conservation and restoration were also present, such as “restoration ecology” (*n* = 40), “reforestation” (*n* = 29), and “conservation” (*n* = 29) (Fig. 3).

**Fig. 4:**
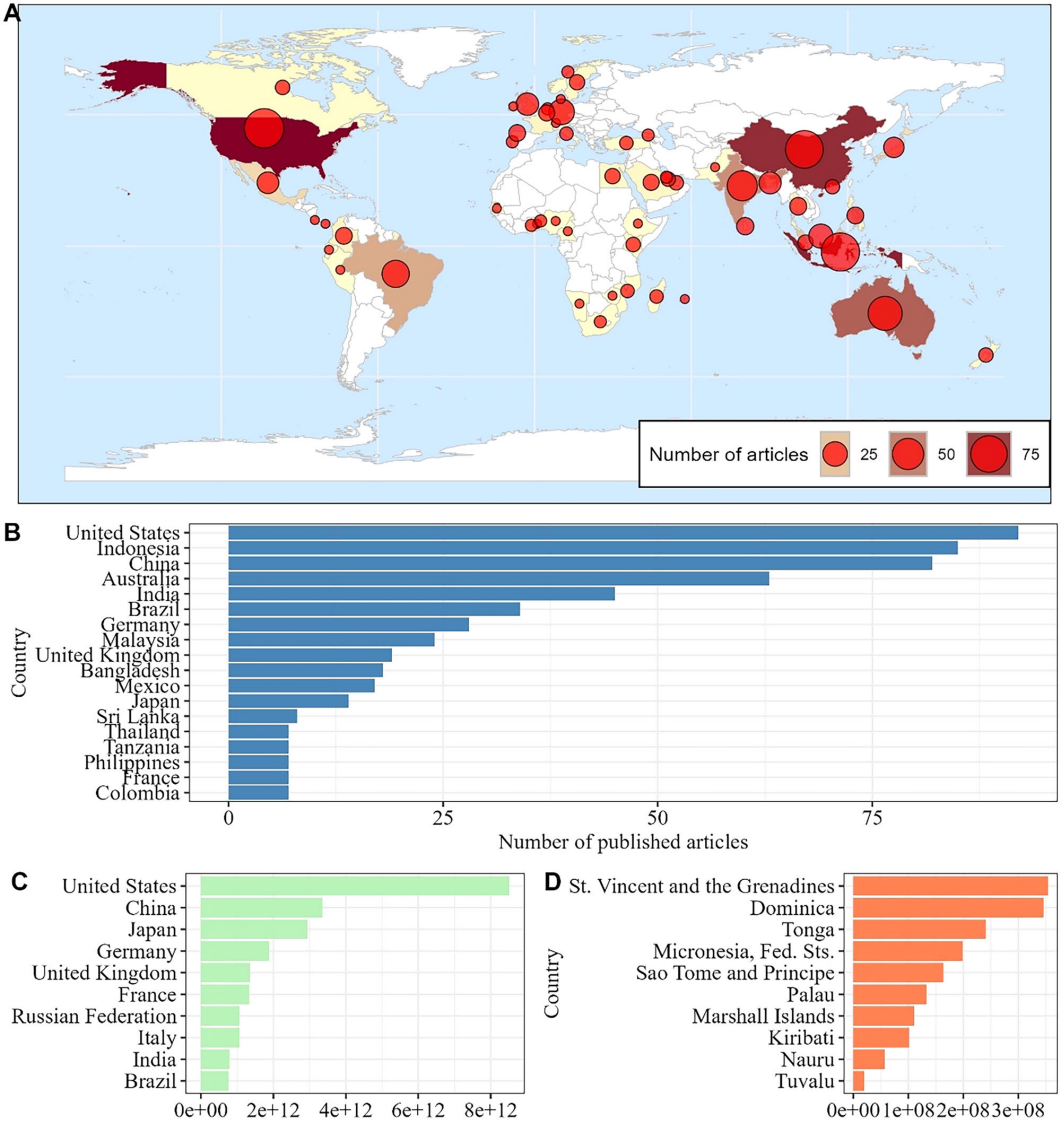
Global distribution of the number of scientific articles published on carbon stocks in riparian forests (**A**), ranking of the top ten countries publishing on carbon stocks in riparian forests (**B**), ranking of countries with the highest (**C**) and lowest (**D**) average Gross Domestic Product (GDP) from 1960 to 2023

A total of 921 articles were published across 61 countries, with the United States having the highest number of included articles (*n* = 92), followed by Indonesia and China, with 85 and 82 articles, respectively. Other countries with significant contributions were Australia (*n* = 63), India (*n* = 45), and Brazil (*n* = 34) (Fig. 4A, B). Additionally, Germany, Malaysia, the United Kingdom, and Bangladesh were also among the top ten publishing countries on the topic (Fig. 4B). Overall, countries classified as part of Global South accounted for most publications (*n* = 667), while Global North contributed 254 articles. Of the 10 countries with the highest average GDP from 1960 to 2023, only two (the United Kingdom and Italy) were not among the 15 most productive in riparian forest carbon research (Fig. 4C). None of most productive countries were among those with the ten lowest GDP in the world (Fig. 4D)

**Fig. 5:**
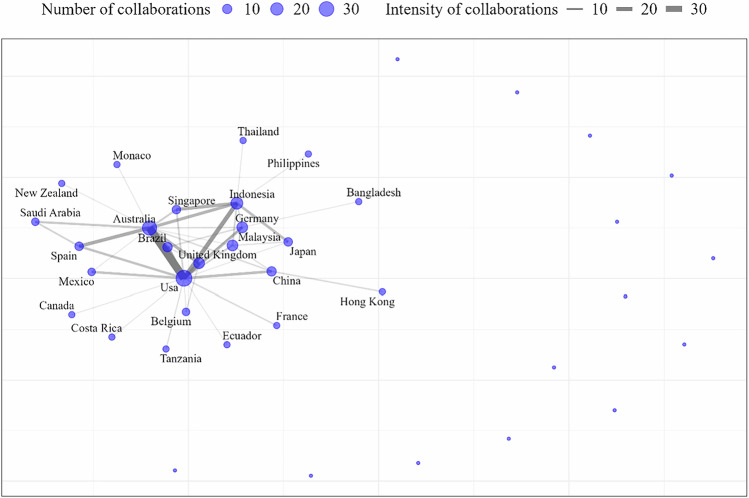
International collaboration based on the institutional affiliations of authors publishing on carbon stocks in riparian forests for countries with more than five collaborations. Point size indicates number of collaborations, and line width represents collaboration strength

The United States led in the number of collaborations, with 471 interactions, followed by Australia (*n* = 331), Indonesia (*n* = 272), the United Kingdom (*n* = 209), Germany (*n* = 184), China (*n* = 182), and Brazil (*n* = 153). Regarding the pairs with the highest frequency of collaboration between countries (Fig. 5), the most frequent partnership was between the United States and Australia (*n* = 76). This was followed by collaborations between the United States and Indonesia, with 21 reciprocal publications, and between the United States and the United Kingdom, with 19 reciprocal publications. Brazil also shows a strong partnership with the United States, accounting for 18 joint publications in each direction. Additionally, cooperation between Australia and Spain totaled 17 bilateral publications (Fig. 5).

Disaster risk from extreme natural events and GDP were associated with changes in the expected number of published articles, whereas life expectancy and literacy rate were not significant (Table 1). The GDP exhibited a positive and statistically significant relationship with scientific output, although IRR = 1.00. Disaster risk from extreme natural events showed a strong positive effect (IRR = 1.0538), indicating that each unit increase in disaster risk is associated with an approximate 5.4% increase in the expected number of publications, suggesting that countries more exposed to climate-related hazards tend to invest more in research on riparian forests and carbon dynamics. In contrast, life expectancy and literacy rate were not significantly related to scientific production. The detailed GLM diagnostics and descriptive analyses are provided in the Supplementary Material.

**Table 1:** Negative binomial regression model predicting the global number of published articles on carbon stocks in riparian forests based on socioeconomic and environmental variables

	Estimate	IRR	Std. Error	*t* value	Pr(>|*t*|)
Intercept	−0.4998	0.6067	1.3080	−0.382	0.7023
GDP (US$ 1.00)	9.24e^−13^	1.0000	2.50e^−13^	3.689	0.0002^*^
Life expectancy (years)	−0.00039	0.9996	0.0325	−0.012	0.9903
Literacy rate (%)	0.0131	1.0132	0.0161	0.813	0.4162
Growth in GHC emissions (%)	0.6350	1.8870	0.3779	1.680	0.0929
Disaster risk from extreme natural events	0.0524	1.0538	0.0115	4.522	6.11e^−6*^

*Estimate* estimated coefficients, *IRR* Incidence Rate Ratio, *Std. Error* standard error, *t value* *t* test values, (*Pr>|t|*) significance values.

*statistical significance at the 5% level.

## Discussion

Riparian forests are strategic ecosystems for biodiversity conservation, water resource protection, and climate regulation due to their capacity to store carbon. Despite this, they remain underestimated in public policies and underrepresented in national carbon inventories, highlighting the need to understand the distribution of scientific production to better inform effective conservation and restoration actions. In this context, the present study provides a comprehensive analysis of the temporal trend of 921 articles published over 27 years, relating it to the geographic distribution and socioeconomic determinants of scientific output on carbon stocks in riparian forests.

Since the signing of the Paris Agreement in 2015, the number of publications increased from 23 in 2015 to 110 in 2023, representing a growth of ~378.26%. From this perspective, international agreements such as the Paris Agreement (2015), the Kyoto Protocol (2005), and the Copenhagen Accord (2009) have driven changes in scientific production over time (Fu and Waltman 2021), as they created demands and highlighted knowledge gaps that must be addressed to underpin public policies on climate mitigation and adaptation. These agreements also emphasized the need for global cooperation (Ourbak and Tubiana 2017), encouraging the alignment of scientific research from various countries with UN resolutions and objectives (Fuentes et al. 2023). Therefore, the increase in scientific production on this topic is closely linked to global governance milestones such as successive IPCC reports, which have emphasized the role of riparian ecosystems as carbon sinks (Mudhee et al. 2025), as well as the Sustainable Development Goals (SDGs), particularly SDG 14 and SDG 6, which address aquatic life conservation and water-related ecosystems (UN 2015).

Moreover, the growth in scientific production and the emergence of the keyword “Remote sensing” among the most used terms from 2011 may be associated with advances in remote sensing technologies, such as LiDAR and high-resolution imagery, which have overcome physical barriers by enabling efficient and cost-effective mapping of these fragmented ecosystems (Bossy et al. 2025). Remote sensing combined with Geographic Information Systems (GIS) tools has facilitated the classification and monitoring of riparian ecosystems using aerial and satellite imagery (Iglseder et al. 2023), enabling the identification of land use and land cover change patterns that may threaten these areas (Sarmiento et al. 2025), as well as assessing riparian ecosystem health (Pace et al. 2022).

The recurrent use of the keywords “carbon sequestration” and “climate change” reflects recognition of riparian forests as relevant ecosystems for studying climate dynamics and carbon stock relationships. Consequently, this underscores the need for the increasing incorporation of forest carbon into national mitigation policies, payment for ecosystem services programs, and mechanisms such as REDD+. Carbon credit policies have rapidly expanded, providing incentives to both private and public sectors to develop innovative approaches aimed at removing carbon from the atmosphere and retaining it in remaining and growing vegetation, especially riparian forests, which, despite being sensitive, tend to grow faster than other ecosystems (Song et al. 2024).

In this context, it was expected that countries with higher GDP would lead research on these topics, as income per capita is commonly associated with national scientific capacity and research output (Jack et al. 2021). However, the geographic distribution of scientific production and GLM results deviate from this expectation, as countries with comparable or higher economic indicators do not necessarily exhibit higher research output, while some lower-income countries show relatively high scientific production. This contrast highlights a paradox when compared to conventional economic indicators (i.e. GDP, and income-related measures), and may reflect global asymmetries in scientific production.

Scientific production on riparian ecosystem services also may be influenced by the geographic distribution and extent of riparian forests themselves. Countries with larger riparian forest areas or extensive river networks may naturally exhibit greater research interest in these systems, independent of their economic indicators. Moreover, dependence on ecosystem services tends to be higher in low-income countries, where local livelihoods are more directly linked to natural resources, although this relationship varies regionally and should be interpreted with caution (Delos et al. 2025). Evidence indicates that, despite experiencing disproportionately negative climate impacts, low-income countries are underrepresented in ecosystem services research, which remains concentrated in high-income regions (Schöngart et al. 2025).

An additional factor that may help explain these patterns is the global geographic distribution of riparian forests themselves, because it are widely distributed across both temperate and tropical regions, but are particularly extensive along large river systems and floodplains, which are unevenly distributed among countries (Dinca et al. 2025). Nations with large territorial extents and dense river networks may therefore harbor larger areas of riparian forests, potentially fostering greater scientific interest and research activity in these ecosystems (Dallaire et al. 2019). Consequently, countries with higher scientific output on riparian forests may not only reflect greater economic or research capacity, but also a greater availability and spatial extent of riparian systems within their territories. Although a global quantification of riparian forest area was beyond the scope of this study, this biophysical context represents an important source of variation that should be considered when interpreting cross-country differences in research output.

In contrast, the significance of the disaster risk from extreme natural events observed in this study highlights the role of environmental vulnerability as an additional and independent driver of scientific attention. Apparently, countries exposed to higher disaster risk tend to prioritize research on ecosystems that provide regulating services, such as riparian forests, which play a key role in flood mitigation, erosion control, and carbon stabilization. In this study, seven of the 15 countries with the highest scientific production on the topic are also among the top 15 countries with the greatest environmental disaster risk, according (Frege et al. 2025): the Philippines (1st); India (2nd); Indonesia (3rd); Colombia (4th); Mexico (5th); China (9th), and Bangladesh (11th). These results suggest that scientific production is not solely a function of economic wealth but is also shaped by exposure to climate-related hazards that generate urgent demands for applied knowledge, monitoring, and adaptation strategies.

Events such as the 2020 droughts in the Brazilian Pantanal (Thielen et al. 2020; Marengo et al. 2021) and the catastrophic floods in Pakistan in 2022 (Chen et al. 2024a, b) demonstrated how hydrological disturbances can release decades’ worth of stored carbon within weeks. Additionally, several low and midle-income countries, particularly in tropical regions, harbor a substancial proportion of remaining natural forests (López**-**Carr 2021). However, it does not necessarily reflect absolute forest area at the global scale, as countries with high GDP such as Russia, Brazil, Canada, and the United States also contain extensive forest cover, but rather differences in forest dependence and land-use dynamics (FAO 2025). In contrast, higher-income countries are often associated with higher CO_2_ emissions due to long-term economic growth and energy demand, particularly where growth relies on non-renewable energy sources (Topa 2024). The combined significance of GDP, World Risk Index and global distribution of scientific production (North and South) therefore reveals a dual mechanism: scientific leadership is maintained by economic capacity of country, while research prioritization is amplified in regions facing higher climate risk. The leadership of the United States was predictable given its consolidated technical capacity and central position in collaboration networks (471 interactions), as was China’s. Furthermore, the collaboration network analysis revealed the centrality of Global North countries, evidencing the persistence of a global knowledge divide and scientific dependency, where institutions from Global North hold symbolic capital to validate research.

Nevertheless, the prominence of countries such as Indonesia (2nd place) and Bangladesh (9th place), which have intermediate GDP per capita but high climate vulnerability (Gan et al. 2021; Jerin et al. 2023), suggests that research efforts are less linked to domestic financial resources and more to immediate environmental pressures and geopolitically driven international cooperation flows. The link between climate vulnerability and research priority has been highlighted by studies showing that regions more susceptible to extreme climate events tend to invest more in applied knowledge for local adaptation and management of essential ecosystem services (Adger 2006; Barua et al. 2020; Huber and Murray 2024). For example, Indonesia became the focus of billion-dollar investments from Norway through REDD+ after identifying its riparian peatlands as critical emission sources, demonstrating how local ecological crises can transform into scientific opportunities through external funding (Firdaus and Arkananta 2024). Moreover, Brazil’s presence among key partners reflects the increasing internationalization of environmental science in the Global South, albeit limited, facing funding instabilities and institutional weaknesses, especially evident in the low valuation of scientific research (Mutinhima et al. 2025).

The absence of significant effects for literacy rate and life expectancy suggests that general social development indicators do not directly translate into increased scientific production on specialized environmental topics. Although these variables are commonly associated with human capital and overall well-being, they may operate indirectly through long-term educational and institutional pathways not captured at the scale of publication count (Uddin et al. 2023). Similarly, the non-significant effect of greenhouse gas emission growth rate indicates that emission trajectories alone are insufficient to stimulate targeted research on riparian forest carbon, particularly when emissions are driven by sectors unrelated to land-use change, such as energy and industry. These results reinforce the interpretation that scientific production in this field is more strongly influenced by structural research capacity and immediate environmental risk than by broad demographic or emission-growth indicators.

From a broader contextual perspective, national policy frameworks may help create enabling conditions for carbon valuation and conservation initiatives. In Brazil, federal programs such as the Sectoral Plan for Mitigation and Adaptation to Climate Change for Consolidating a Low-Carbon Economy in Agriculture (Plano ABC) and its updated version (ABC+) support rural producers in recovering degraded areas, integrating crop–livestock–forestry systems, and conserving riparian forests (Brasil 2009). Complementary strategies, including the National Payment for Environmental Services Program and the Floresta+ Program, further promote conservation and restoration through financial incentives (Brasil 2021). In addition, Brazilian legislation, notably the Forest Code, mandates the maintenance of Permanent Preservation Areas (APPs) and Legal Reserves, providing an institutional framework for riparian forest protection (Brasil 2012).

Voluntary carbon credit markets have been strengthening in Brazil as alternatives to attract private investments in restoration and sustainable management projects, fostering a new bioeconomy dimension (Araujo et al. 2024). This strategy can create opportunities for governments, companies, and communities to act integrally, transforming conservation into an income-generating activity that provides tangible benefits to rural populations dependent on natural resources (Stephenson and Damerell 2022). In this context, the role of intermediaries such as international funding agencies, NGOs, and universities with strong involvement in transnational networks is indispensable and should be valued to ensure progress in studies on carbon in riparian ecosystems.

## Conclusion

Over the past two decades, scientific production has grown significantly, coinciding with technological advances and global environmental policies such as REDD+ and carbon markets. However, the results of this study indicate that traditional economic indicators generally show weak explanatory power for scientific output. Despite that, GDP emerged as a statistically significant predictor, although its effect size was modest. Significative association with disaster risk from extreme natural events revealed that environmental vulnerability is a significant driver of scientific production, highlighting that research effort is also shaped by exposure to climate-related hazards and ecosystem sensitivity. Therefore, scientific output appears to be governed by a dual mechanism, combining structural research capacity, represented by GDP, and contextual environmental pressure, represented by disaster risk. This highlights the importance of strengthening scientific infrastructure and research capacity in emerging and vulnerable countries, while also fostering international scientific cooperation to address global knowledge gaps.

## Data Availability

No datasets were generated or analysed during the current study.
